# Effectiveness of herpes zoster vaccination in an older United Kingdom population

**DOI:** 10.1016/j.vaccine.2018.02.021

**Published:** 2018-04-19

**Authors:** Jemma L. Walker, Nick J. Andrews, Gayatri Amirthalingam, Harriet Forbes, Sinead M. Langan, Sara L. Thomas

**Affiliations:** a Statistics, Modelling and Economics Department, Public Health England, 61 Colindale Avenue, London NW9 5EQ, UK; b Faculty of Epidemiology and Population Health, London School of Hygiene and Tropical Medicine, Keppel Street, London, UK; c Immunisation, Hepatitis and Blood Safety Department, Public Health England, 61 Colindale Avenue, London, UK

**Keywords:** Herpes zoster, Vaccine, Effectiveness, General practice, Electronic health records, United Kingdom

## Abstract

**Background:**

Vaccination against herpes zoster was introduced in the United Kingdom in 2013 for individuals aged 70 years, with a phased catch-up campaign for 71–79 year olds. Vaccine introduction has resulted in a marked fall in incident herpes zoster and in post-herpetic neuralgia (PHN), but formal evaluation of vaccine effectiveness is needed.

**Methods:**

In a population-based cohort study of older individuals born between 1933 and 1946, we used linked UK anonymised primary care health records for the first three years of the vaccination programme (01/09/2013–31/08/2016) and multivariable Poisson regression to obtain incidence rates and vaccine effectiveness (VE) against zoster and PHN.

**Results:**

Among 516,547 individuals, 21% were vaccinated. Incidence of zoster was 3.15/1000 person-years in vaccinees and 8.80/1000 person-years in unvaccinated individuals. After adjustment, VE was 64% (95%CI = 60–68%) against incident zoster and 81% (95%CI = 61–91%) against PHN, with very similar VE estimates in the routine and catch-up cohorts. VE against zoster was lower in those with a previous history of zoster: 47% (95%CI = 31–58%) versus 64% (95%CI = 60–68%) in those without previous zoster. There was evidence of waning VE over time, from 69% (95%CI = 65–74%) in the first year after vaccination to 45% (95%CI = 29–57%) by the third year.

**Conclusion:**

This first formal assessment of VE in the UK zoster vaccination programme demonstrates good effectiveness of zoster vaccine, and very good protection against PHN. The findings provide evidence that VE is similar across the age groups targeted for vaccination in the UK, and on duration of protection of the vaccine in public health use. The study provides key information for decision-makers about the future direction of UK zoster vaccination programme, indicating that the live zoster vaccine may be more cost-effective than estimated previously. It also supports efforts to communicate the benefits of zoster vaccination to address the declining coverage observed across the UK.

## Highlights

• Live zoster vaccine had good vaccine effectiveness (VE) in UK public health use.
• VE against incident zoster was 64%, and against post-herpetic neuralgia was 81%.
• VE was very similar in the two age groups targeted for vaccination.
• VE was lower (47%) among those with history of zoster.
• The UK zoster vaccine programme may be more cost-effective than previous estimates.

## 1 Introduction

Herpes zoster occurs following reactivation of latent varicella zoster virus (VZV) infection. It results in a painful unilateral dermatomal rash and appreciable short- and long-term morbidity, notably prolonged pain (post-herpetic neuralgia, PHN). Reactivation of VZV as zoster is prevented by specific cell-mediated immunity, and thus those who are immunosuppressed are at increased zoster risk [1]. Zoster incidence also rises markedly with age, due to immunosenescence and loss of specific immunity to VZV, with rates of 8–12 per 1000 person-years in individuals aged 80+ years [2], [3]. Older individuals are also more likely to develop PHN following zoster [4].

In 2006, a live attenuated vaccine against zoster (Zostavax; Zoster Vaccine Live; Merck & Co) was licensed for use and introduced in the USA for older individuals. The large US pre-licensure trial of the vaccine in individuals (median age: 69 years) followed up for a mean of 3.13 years demonstrated vaccine efficacy against incident zoster of 51%, with 67% efficacy against PHN [5]. Subsequent US post-licensure studies of older individuals, with maximum follow-up of 2–5.8 years, have reported estimates of vaccine effectiveness (VE) against incident zoster [6], [7], [8], [9], [10], [11]. In those with estimates in the first three years of follow-up VE has varied from 33% to 55% [6], [7], [9]. Three post-licensure studies also reported VE against PHN, with estimates of 55% and 59% in the first three years [6], [9] and 61% in a case-control study which included individuals vaccinated up to 4.8 years before their zoster diagnosis [10].

Zoster vaccination was introduced in the UK in September 2013, targeted at individuals aged 70 years (the routine cohort) on 1st September of that year, and those aged 70 years on 1st September 2014 and 1st September 2015 in the second and third year of the programme [12], [13]. There was also a phased introduction of a catch-up campaign for older individuals, with the vaccine offered to those aged 79 years on 1st September 2013 (in the first year); 78 and 79 years on 1st September 2014 (second year), and 78 years on 1st September 2015 (third year). For both the routine and catch-up cohorts, unvaccinated individuals continued to be eligible for vaccination until their 80th birthday [12]. As a live vaccine, the vaccine is contraindicated for individuals with specific immunosuppressive conditions or receiving immunosuppressive therapy as defined in national guidance [12]. The vaccine is offered in general practice throughout the year, although practices are encouraged to administer it alongside the annual seasonal influenza vaccination programme. Vaccine uptake in England has declined over time, from 61.8% (first year) to 54.9% (third year) in the routine cohort, and from 59.6% to 55.5% in the catch-up cohort [14], [15].

We recently showed that general practice consultations for zoster and for PHN in the first three years of the programme decreased by 35% and 58% respectively in the routine cohorts, and by 33% and 38% in the catch-up cohorts [16]. However, this ecological study assessed vaccine impact and did not use patient-level data to formally estimate VE. Therefore, in this study we used linked individual-level electronic health records to estimate effectiveness of the zoster vaccine in the first three years of the UK programme against incident zoster and PHN.

## 2 Methods

### 2.1 Data sources

We used anonymised data from the Clinical Practice Research Datalink (CPRD), which comprises the primary care records from a representative 7% sample of the UK population [17]. Approximately 58% of the practices have consented to taking part in the CPRD linkage scheme, and patients’ records from these practices are linked to hospitalisation data (Hospital Episode Statistics, HES), and to patient-level deprivation data (Index of Multiple Deprivation for the postcode of the patient’s residence) [17].

### 2.2 Study population

We selected individuals who had at least one year’s prior registration with a CPRD practice on 1st September 2013. For reasons of confidentiality, CPRD patients’ month and day of birth are not available to researchers. We therefore chose individuals born in the years 1933–1946, to ensure that we included those eligible for vaccination in the routine programme (individuals born 2/9/1942–1/9/1945, aged 68–70 years in September 2013) and those eligible for the catch-up programme (born 2/9/1933–01/09/1937, aged 76–79 in September 2013). Inclusion of the remaining individuals in the study added statistical power for determining age effects and extra person-time. In addition, including unvaccinated individuals who were ineligible (not of eligible age) for vaccination helped to mitigate potential confounding by health-seeking behaviour. This is because our unvaccinated group comprised not only those who were eligible for vaccination but did not come forward (who may be at different risk of zoster to eligible individuals who accepted vaccination), but also those who were similar in age but ineligible for vaccination (who would be less likely to be at different baseline risk of zoster to vaccinated individuals).

We excluded from the study population those who had no contact with their practice (a consultation or prescription) or other evidence of ongoing care (e.g. recording of test results) at any time from one year prior to 1st September 2013 until the end of the follow-up, to remove individuals who were inactive in the database. We also excluded those who had received zoster vaccine before the start of the national vaccination campaign, and those who at any point during the study period had an immunosuppressive condition or therapy that was a contraindication for zoster vaccination (Appendix A details how these conditions/therapies were identified).

### 2.3 Outcomes

Incident zoster diagnoses were identified using Read codes in the CPRD data, supplemented in those with linked hospitalisation data by diagnoses identified using ICD-10 (International Classification of Diseases, 10th revision) codes in the primary or secondary diagnosis fields of a hospitalisation (code lists are in Appendix B). If zoster codes occurred in both the CPRD and HES data, the earlier code was taken as the incident date. Individuals with ongoing zoster episodes at the start of the study period were not considered at risk of zoster for 365 days after their zoster incident date, or (for individuals with ongoing zoster consultations extending beyond a year) until 90 days after their last zoster code. Episodes beginning with a PHN code were also not included in analyses, as we could not ascertain whether these individuals had past zoster with ongoing PHN rather than an incident zoster episode; these individuals also started follow-up at the end of the episode.

We defined PHN as pain persisting ≥3 months after the zoster diagnosis [5]. Although there are specific Read and ICD-10 PHN codes, general practitioners often do not use them, instead recording consultations for pain or prescribed pain medications. We therefore used an update of our PHN algorithm, based on a validated algorithm developed for US administrative health data [18], [19]. Briefly, we looked in the 90–365 days after the zoster diagnosis for evidence of: PHN codes; combinations of zoster codes, neuralgia codes and medications used to treat PHN; and referrals to pain clinics (full definitions are in Appendix C). As anticonvulsant drugs and codes for neuropathic pain/neuralgia comprised part of our PHN algorithm, we excluded from the PHN analysis patients with a history of epilepsy, other specific conditions that cause neuropathic pain, or trigeminal neuralgia. We also restricted patients to those with at least 365 days follow-up after a zoster diagnosis (or, for those who did not develop zoster, at least 365 days follow-up in the study period) to enable full assessment of PHN.

### 2.4 Vaccination status

This was determined from patients’ immunisation, clinical, therapy and referral files in CPRD. Those with conflicting vaccination status on the same day (for example records indicating that the vaccine was both refused and given) were dropped from our study. Patients were considered fully vaccinated 42 days after the vaccine was given.

### 2.5 Covariates

A priori confounders of the relationship between vaccination status and the outcomes of interest included age, study year and calendar month. Further potential confounders included sex, ethnicity, deprivation status, geographic region, previous history of zoster, and uptake of seasonal influenza vaccination in the previous year (as a marker of propensity to consult, and because zoster vaccine was offered alongside influenza vaccine). We also considered co-morbidities that are risk factors for zoster, namely rheumatoid arthritis, systemic lupus erythematosus, inflammatory bowel disease, chronic kidney disease, chronic obstructive pulmonary disease, asthma and diabetes mellitus [20]. These conditions were identified using the Read code lists from our previous zoster study, with individuals considered to have the condition if they had a diagnostic code at any time before the start of the study [20]. Age was also grouped to capture the routine (born 1940–1946) and catch-up (born 1933–1939) cohorts.

### 2.6 Statistical methods

Start of follow up was the later of 1st September 2013 (the date of zoster vaccination introduction) and the end of any previous zoster episode that occurred prior to September 2013. End of follow up for the zoster incidence analysis was the earliest of the date the patient left the practice or died, the date the data were last collected from the practice, the date of the first zoster episode, and 31st August 2016. For PHN analyses, we only considered zoster episodes that started on or before 31st Aug 2015, to allow full assessment of PHN in the year after the zoster diagnosis. The incident zoster date was used as the date of incident PHN, thus assessing VE against zoster that resulted in PHN.

Vaccine effectiveness against both zoster and PHN was assessed using multivariable Poisson regression with an offset for person-time to obtain adjusted incidence rate ratios (IRR). Effectiveness was calculated as (1−IRR) ∗ 100. The IRRs were adjusted for the *a priori* confounders year of age (modelled first as a linear and then as a quadratic effect), year of study and calendar month; other potential confounders were retained in the model if they changed the IRR by ≥5%. We added interaction terms to the models to investigate whether VE varied by sex, age (routine vs catch-up cohort), or between those with and without a history of zoster. Effectiveness was also calculated by time since vaccination, to examine waning of protection.

All analyses were carried out using Stata MP 14.

### 2.7 Ethics approval

Approval was received from the Independent Scientific Advisory Committee of the Medicines and Healthcare Products Regulatory Agency (ISAC number: 13.138RA) and the Ethics Committee of the London School of Hygiene & Tropical Medicine (Reference number: 14643).

## 3 Results

**Fig. 1:**
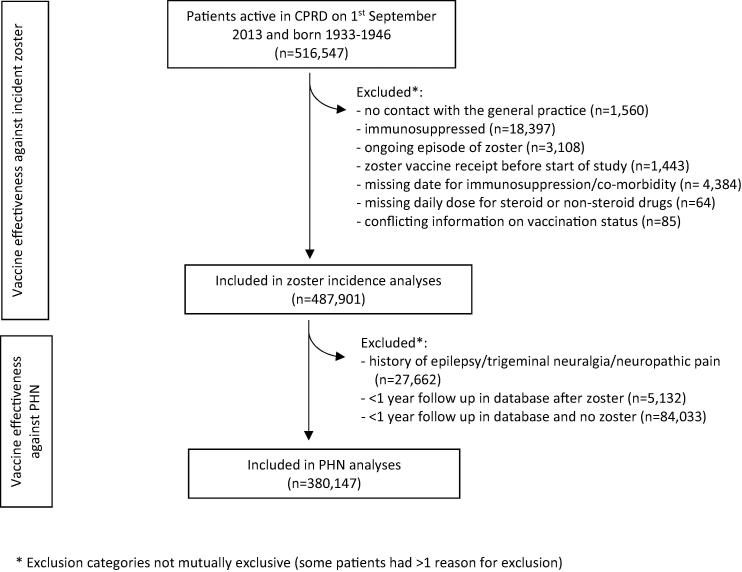
Selection of individuals for vaccine effectiveness analyses.

Among 516,547 patients born between 1933 and 1946 and active in CPRD on 1st September 2013, 487,901 individuals with 1,059,179 person-years of follow up (excluding the 42 days of person-time after vaccination) were eligible for inclusion in the analyses of VE against incident zoster (Fig. 1). Of these, 380,147 individuals with 585,540 person-years of follow up were included in analyses of effectiveness against PHN. The characteristics of the study population are summarised in Table 1. Vaccinated individuals in the zoster incidence analyses were followed up for a median of 1.42 person-years (range: 0.003–2.9), and unvaccinated individuals were followed up for a median of 1.99 person-years (range: 0.003–3.0).

**Table 1:** Person-years by vaccination status and study population characteristics for (a) zoster incidence analyses and (b) post-herpetic neuralgia (PHN) analyses.

Characteristic	(a) Zoster incidence analyses person years:^a^		(b) PHN analyses person years:^a^	
	Vaccinated^b^ (137,968py)	Unvaccinated^b^ (909,474 py)	Vaccinated^c^ (52,861py)	Unvaccinated^c^ (526,055py)
*Sex*				
Male	64,925	424,381	24,472	242,812
Female	73,044	485,093	28,389	283,243

*Time since vaccination*				
Unvaccinated	–	909,474	–	526,055
Up to 1 year since vaccination	75,707	–	39,172	–
1 to <2 years since vaccination	47,954	–	13,689	–
2–3 years since vaccination	14,307	–	0^4^	–

*Ethnicity*				
White	81,378	527,387	30,286	297,068
South Asian	1275	11,318	436	5586
Black	615	6751	240	3428
Other	580	5617	212	3093
Mixed	149	1277	55	672
Not stated	6480	46,281	2,512	26,740
Missing	47,492	310,843	19,120	189,467

*Cohort*				
Routine (born 1940–1946)	77,319	540,007	30,631	313,448
Catch-up (born 1933–1939)	60,649	369,467	22,230	212,606

*IMD status*				
1	19,903	127,120	7475	71,164
2	16,305	115,308	5849	62,392
3	14,603	104,251	5336	57,082
4	10,813	81,814	3879	44,049
5	6772	54,584	2463	28,871
Missing	69,573	426,397	27,859	262,497

*History of zoster*				
History	14,784	88,358	5753	51,730
No history	123,185	821,116	47,108	474,325

*Influenza vaccination*				
Given the previous year	127,358	653,297	48,569	379,111
Not given the previous year	10,610	256,177	4292	146,943

a Excludes the person-time in the 6 weeks after vaccination.

b Median follow-up time: vaccinated = 1.42 person-years (range: 0.003–2.9); unvaccinated = 1.99 person-years (range: 0.003–3.0).

c Median follow up time: vaccinated = 0.80 person-years (range: 0.003–1.88); unvaccinated = 1.69 person-years (range 0.003–2.0).

d Only includes follow-up of zoster to the end of year 2, to allow detection of PHN in the year after incident zoster.

A total of 103,336 individuals (21% of the study population, 15.2% of person-time) were vaccinated. During the study period, 435 vaccinated individuals developed zoster >42 days after vaccination, compared to 8006 unvaccinated individuals, resulting in zoster incidences of 3.15/1000 person-years and 8.80/1000 person-years respectively (Table 2). After adjusting for age, month and year of the study, overall VE against incident zoster was 64% (95%CI = 60–68%). Further adjustment by sex, geographic region, comorbidities, previous history of zoster and previous receipt of influenza vaccination, and (in complete case analyses) ethnicity and IMD did not change the effectiveness estimate (data not shown). Adding age as a quadratic variable made no material difference to the results. As 31% of individuals were missing information on ethnicity, we repeated analyses restricted to those of White ethnicity; effectiveness was identical to that found for the entire cohort (VE = 64%, 95%CI = 60–69%).

**Table 2:** Vaccine effectiveness (VE) against (a) incident zoster and (b) post-herpetic neuralgia (PHN).

Vaccination status	Cases (n)	Person-years^a^	Rate/1000py	Adjusted IRR^b^ (95% CI)	VE (95% CI)
*(a) Outcome: incident zoster*					
Unvaccinated	8006	909,474	8.80	1.00	1.00
Vaccinated	435	137,968	3.15	0.38 (0.34, 0.42)	62% (58%, 66%)
*(b) Outcome: PHN*					
Unvaccinated	371	526,055	0.70	1.00	
Vaccinated	8	52,861	0.15	0.19 (0.09, 0.39)	81% (61%, 91%)

a Excludes person-time in the 42 days after vaccination.

b Incidence rate ratio, adjusted for age in years, month and year of study.

There was no evidence that VE differed in the routine and catch-up cohorts (p_interaction_ = 0.58), and only weak evidence that VE was lower among women (VE = 60% (95%CI = 55–64%) versus VE = 66% (95%CI = 60–71%) among men, p_interaction_=0.08, Table 3). In contrast, VE was lower among those with a history of zoster (VE = 47%, 95%CI = 31–58% versus VE = 64%, 95%CI = 60–68% among those with no history of zoster, p_interaction_ = 0.009, Table 3). Stratification by time since vaccination showed evidence of waning, with VE decreasing from 69% (95%CI = 65–74%) in the first year after vaccination to 45% (95%CI = 29–57%) in the third year (p_interaction_ < 0.001, Table 3). This waning was seen in both age cohorts, with VE in the routine cohort falling from 71% in the first post-vaccination year to 52% by the third year, and VE in the catch-up cohort falling from 69% to 38% (Table 3).

**Table 3:** Vaccine effectiveness (VE) against incident zoster by sex, vaccination cohort, history of zoster and time since vaccination.

Factor	Vaccine effectiveness against incident zoster				
	Level	Vaccinated cases/Pyears^a^ (rate)^b^	Unvaccinated cases/Pyears^a^ (rate)^b^	VE (95% CI)^c^	p_interaction_^d^
By vaccination cohort	Routine	230/77,319 (2.97)	4521/540,007 (8.37)	62% (56%, 67%)	
	Catch-up	205/60,649 (3.38)	3485/369,467 (9.43)	64% (58%, 69%)	0.58^e^
By sex	Male	161/64,925 (2.48)	3271/424,381 (7.71)	66% (60%, 71%)	
	Female	274/73,044 (3.75)	4735/485,093 (9.76)	60% (54%, 64%)	0.08^e^
By history of zoster	No history	365/123,185 (2.96)	7145/821,116 (8.70)	64% (60%, 68%)	
	History	70/14,784 (4.73)	861/88,358 (9.74)	47% (31%, 58%)	0.009^e^
Time since vaccination	<=1 year	197/75,707 (2.60)		69% (65%, 74%)	
	1–2 years	178/47,954 (3.71)	8006/909,474 (8.80)	54% (47%, 61%)	
	2–3 years	60/14,307 (4.19)		45% (29%, 57%)	<0.001^f^
Time since vaccination by cohort	Routine				
	<=1 year	95/41,602 (2.28)		71% (65%, 76%)	
	1–2 years	103/26,640 (3.87)	4521/540,007 (8.37)	49% (38%, 58%)	
	2–3 years	32/9077 (3.53)		52% (32%, 67%)	0.0002^f^
	Catch-up				
	<=1 year	102/34,105 (3.00)		69% (62%, 74%)	
	1–2 years	75/21,313 (3.52)	3485/369,467 (9.43)	61% (50%, 69%)	
	2–3 years	28/5230 (5.35)		38% (9%, 58%)	0.01^f^

a Excludes person-time in the 42 days after vaccination.

b Rate/1000 person-years.

c Adjusted for age in years, month and year of study.

d p value for interaction.

e Calculated using Wald test for interaction.

f Calculated using likelihood ratio test.

Eight cases of PHN occurred in vaccinated individuals >42 days after vaccination and 371 cases occurred in unvaccinated individuals, After adjusting for age, month and year of the study, VE against PHN was 81% (95%CI = 61–91%, Table 2). As with the zoster incidence analyses, effectiveness against PHN was similar in the routine and catch-up cohorts (routine: VE = 84%, 95%CI = 50–95%; catch-up: VE = 79%, 95%CI = 48–92%). There was no statistical evidence of heterogeneity in VE by sex, history of zoster, or time since vaccination (p_interaction_ > 0.4 for all), but analyses were limited by the small number of PHN cases among vaccinated individuals.

## 4 Discussion

This first formal assessment of VE in the UK zoster vaccination programme provides important evidence of the effectiveness of zoster vaccine in public health use, demonstrating 62% effectiveness against incident zoster and higher effectiveness (81%) against its most common severe complication, PHN. We found very similar VE in the two age groups targeted for vaccination in the UK, but lower effectiveness (47%) among those with a previous history of zoster. Effectiveness decreased within the first three years after vaccination, from 69% in the first year to 45% by three years.

Our findings add to our recent ecological study of the impact of vaccine introduction in the UK, which showed marked reductions in both zoster incidence and PHN in the first three years of the vaccination programme [16]. Our current study’s VE estimates are somewhat higher than those from the US pre-licensure trial(which included individuals aged ≥60 years at enrolment, with >6.5% older than 80 years): 62% vs 51% against incident zoster and 81% vs 67% for PHN [5]. However, the cases in the current study all sought care for their zoster, whereas the trial involved active follow up of all participants. We therefore may not have captured the mildest cases of zoster, and our findings are consistent with reports that the vaccine may afford higher protection against more severe zoster [5], [9]. Furthermore, the confidence intervals for the two PHN VE estimates overlapped, and the differences could reflect random error. Our finding that VE was near-identical in the routine and catch-up cohorts is also different to the trial results, but consistent with findings from two US post-licensure studies that reported very similar VE in those aged 70–74 years and 75–79 years [7], [9]. Two recent US studies that stratified VE estimates by both age and time since vaccination have also shown similar waning of VE in the first, second and third years, with VE of 64.5%, 45.2% and 36.8% among individuals aged 70–79 years, and 67%, 47% and 34% among individuals aged ≥70 years [8], [11]. The lower VE we identified among those with a history of zoster is, to the best of our knowledge, a novel finding that warrants further investigation. Those who experience a second zoster event may have less well-functioning immune systems (among whom the vaccine may not be as effective). However, we had no information on the timing of past zoster, which could explain our results if there was differential uptake among those with recent and less recent zoster – for example if those who had more recent zoster (who might be well protected against subsequent VZV reactivation because of boosted immunity) were less likely to come forward for vaccination compared to those who had zoster less recently (who would be less well protected, due to waning immunity).

Our study has several strengths. This was a large, population-based study using a representative sample of the UK population in a general practice setting; as the UK zoster vaccine programme is delivered via general practice, this maximised capture of zoster vaccination status. Linkage to hospitalisation records enabled better ascertainment of zoster, including cases that presented directly to hospital that might be incompletely recorded in the general practice data. Our detailed algorithm for identifying PHN allowed identification of cases that would have been missed if we had restricted the PHN case definition to specific PHN medical codes. Similarly, we used extensive methods to identify and exclude individuals with immunosuppression who are ineligible to receive the live zoster vaccine. As these individuals should almost all be unvaccinated and are at appreciably higher risk of zoster, their exclusion from analyses helped to avoid underestimation of VE. Despite not having patients’ exact date of birth (and thus not pinpointing exactly those eligible for vaccination), inclusion of a range of birth cohorts may have helped to minimise confounding resulting from health-seeking behaviour.

Some study limitations also need consideration. Zoster is usually diagnosed clinically, without virological confirmation. Thus, misclassification of the outcome may have occurred. However, zoster has a very characteristic clinical presentation and the diagnosis is usually straightforward. A Dutch validation study of zoster diagnoses made in primary care reported that nearly 91% of individuals with clinical zoster had elevated VZV antibodies (suggesting recent reactivation of VZV), demonstrating a high positive predictive value for the clinical diagnosis by general practitioners [21]. Despite our extensive range of case definitions used to identify PHN, we may have not ascertained some cases, due to the variety of ways in which general practitioners can record these diagnoses. Furthermore, some individuals with persistent pain after zoster may have used over-the-counter pain medications and not consulted their GP; as all these individuals sought care for their initial zoster, it is likely that any misclassification would be non-differential with respect to vaccination status, resulting in possible under-estimation of VE against PHN. Despite the large size of our data, there were few PHN events among vaccinated individuals, decreasing the precision of some estimates and preventing investigation of effect modification. A further potential limitation of the PHN analysis was the requirement that individuals have at least one year follow-up after zoster (for those who developed zoster) or one year active follow-up (for those who did not develop zoster), to allow sufficient time to capture PHN. This additional inclusion criterion may have limited the generalisability of our VE estimate to those who did not die soon after developing zoster. However, as less than 2% of individuals (both those with zoster and those without) who were excluded from the PHN analyses were excluded because they had died, this is unlikely to have affected the generalisability of our findings to any great extent. We considered many potential confounders including a range of comorbidities associated with increased zoster risk, but residual confounding could have occurred, for example if more frail individuals (with less well-functioning immune systems) were less likely to receive zoster vaccination. Information on participants’ ethnicity was incomplete, but the results of our sensitivity analysis restricted to individuals of White ethnicity suggests that this did not affect results.

These new VE estimates, including the evidence of waning over time, contribute key information for decision-makers about the UK zoster vaccination programme. Our finding of similar VE among younger and older individuals is important, given the choice of age group currently offered vaccination (those aged 70 and 78/79 years). Reviewing optimal vaccine strategies also includes consideration of the relative merits of the current live vaccine with the new sub-unit zoster vaccine, which (unlike the live vaccine) can be administered to those with immunosuppression, a group at particularly high risk of zoster [22]. Notably, our VE estimates indicate that the live zoster vaccine programme might be more cost-effective for the NHS than estimated in the 2009 study which modelled VE based on the results from the US pre-licensure trial [23]. Ongoing monitoring of zoster VE over time will be important to obtain robust estimates of waning effectiveness over a longer period. Our findings should also help to demonstrate to healthcare workers and the older population that the vaccine is effective, particularly against long-term pain following zoster. This may help to increase vaccination coverage among the older UK population and reverse the recent decline in vaccine uptake.

## Author contributions

GA and NA conceived the study. NA, ST, JW, and GA designed the study, with input from HF and SL. ST carried out the literature search. JW analysed the data. JW and ST drafted the paper and all authors were involved in the revision and approval of the final content before submission.

## Funding

This research was funded by the National Institute for Health Research Health Protection Research Unit (NIHR HPRU) in Immunisation at the London School of Hygiene and Tropical Medicine in partnership with Public Health England (PHE). SML is supported by a Wellcome senior research fellowship in clinical science (205,039/Z/16/Z). The views expressed are those of the authors and not necessarily those of the NHS, the NIHR, the Department of Health, the Wellcome Trust or Public Health England. The funders had no role in the study design, data collection, analysis or interpretation of the data, in the writing of the manuscript or in the decision to submit the paper for publication.

## Conflict of interest

The Immunisation, Hepatitis and Blood Safety Department of Public Health England has provided vaccine manufacturers with post-marketing surveillance reports which the Marketing Authorisation Holders are required to submit to the UK Licensing Authority in compliance with their Risk Management Strategy. A cost recovery charge is made for these reports, which have not to date included herpes zoster. JW, NA, GA, HF, SL and ST have no other reported conflicts.
