# Supplementary material for: Effectiveness of herpes zoster vaccination in an older United Kingdom population
Source: Vaccine. 2018 Apr 19;36(17):2371–7. doi: 10.1016/j.vaccine.2018.02.021 (PMC5899761; doi:10.1016/j.vaccine.2018.02.021)
Supplement: Supplementary data 1 [file mmc1.docx]

**Appendix A:** **Criteria used to ascertain immunosuppressive conditions and treatments**

The methods used to identify immunosuppressive conditions and therapies that are contraindications of zoster vaccination uptake were adapted from our earlier study of risk factors of herpes zoster.^19^ Immunosuppressive conditions included: leukaemia, lymphoma, myeloma, other plasma cell dyscrasias, stem cell and bone marrow transplants, solid organ transplants, Human Immunodeficiency Virus infection and cellular immune deficiency. The immunosuppressive treatments included were: methotrexate, azathioprine, 6-mercaptopurine, biological therapies, steroids, other immunosuppressive agents (e.g. tacrolimus), other disease modifying anti-rheumatic drugs (e.g. ciclosporin), chemotherapy and radiotherapy. We used data on the number of tablets prescribed and the numeric daily dose to calculate the duration of the prescription. As with our previous study, we imputed missing numeric daily dose data, using a “hot-desk” style imputation method.^19^ The code lists used to identify these conditions and therapies are available on request.

The Table below summarises the immunosuppressive dose criteria and the duration of immunosuppression used for each condition/treatment.

| **Immunosuppressive condition or therapy** | **Dose criteria if applicable** | **Period of immune-suppression prior to first medical record** | **Period of immunosuppression after each record** |
| --- | --- | --- | --- |
| Lymphoma, myeloma, other plasma cell dyscrasias, leukaemia, bone marrow transplant stem cell transplant | N/A | - | 24 months |
| Cellular immune deficiency, solid organ transplants and HIV | N/A | - | For life |
| Azathioprine | Immunosuppressed at a dose of ≥50mg/day | 3 months^1^ | 3 months |
| Methotrexate | Immune-suppressed at dose of>25mg per week (>3.57 mg/day) | 3 months^1^ | 3 months |
| 6-mercaptopurine | Immunosuppressed at a dose of ≥45mg/day | 3 months^1^ | 3 months |
| Other immunosuppressive agents | Immunosuppressed at any dose | 3 months^1^ | 3 months |
| Biological agents (e.g. Anti-TNF therapy) | Immunosuppressed at any dose | 3 months^1^ | 12 months |
| Other disease-modifying anti-rheumatic drugs e.g. mycophenolate, leflunomide | Immunosuppressed at any dose | 3 months^1^ | 3 months |
| Injectable or oral steroids | Immunosuppressed at >40 mg/day for >7 days, or at >20mg/ day for >14 days | 3 months^1^ | 3 months |
| Cancer chemotherapy or radiotherapy | Immunosuppressed at any dose | 3 months^1^ | 12 months |

^1^3 month period before the first general practice prescription was added as the therapy is typically initiated in hospital

Appendix B: Zoster and post-herpetic neuralgia codes in CPRD and HES

**Zoster codes in CPRD**

| **Medical code** | **Read term** |
| --- | --- |
| 390 | Herpes zoster |
| 516 | Shingles |
| 7331 | Ramsey Hunt Syndrome |
| 8936 | Ophthalmic herpes zoster infection |
| 14718 | Herpes zoster with ophthalmic complication |
| 14793 | Herpes zoster otitis externa |
| 18918 | Herpes zoster ophthalmicus |
| 21069 | Herpes zoster with unspecified complication |
| 21471 | Herpes zoster NOS |
| 25320 | Herpes zoster with dermatitis of eyelid |
| 27403 | Geniculate herpes zoster |
| 27546 | Herpes zoster with keratoconjunctivitis |
| 31681 | Herpes zoster - otitis externa |
| 33810 | Herpes zoster with other ophthalmic complication |
| 38531 | Herpes zoster with other specified complication NOS |
| 39692 | Polyneuropathy in herpes zoster |
| 43235 | Herpes zoster with other specified complication |
| 44944 | Herpes zoster with meningitis |
| 47375 | Zoster encephalitis |
| 50537 | Herpes zoster with other CNS complications |
| 51692 | Encephalitis due to herpes zoster |
| 52126 | Herpes zoster with other central nervous system complication |
| 52319 | Disseminated zoster |
| 55940 | Herpes zoster iridocyclitis |
| 57895 | Herpes zoster meningitis |
| 62558 | Infective otitis externa due to herpes zoster |
| 63739 | Herpes zoster with other CNS complication NOS |
| 69405 | Herpes zoster encephalitis |
| 70197 | [X]Zoster without complications |
| 71464 | Meningitis due to herpes zoster virus |
| 105157 | Hutchinson's sign - herpes zoster involving nose tip |

**Post-herpetic neuralgia codes in CPRD**

| **Medical code** | **Read term** |
| --- | --- |
| 1598 | Post-herpetic neuralgia |
| 7584 | Post-herpetic trigeminal neuralgia |
| 10223 | Postherpetic neuralgia |
| 17180 | Postzoster neuralgia |
| 31709 | Postherpetic polyneuropathy |
| 11498 | Postherpetic trigeminal neuralgia |

**Zoster codes in HES**

| **ICD code** | **ICD description** |
| --- | --- |
| B02 | Herpes zoster |
| B02.0 | Zoster encephalitis |
| B02.1 | Zoster meningitis |
| B02.3 | Zoster ocular disease |
| B02.7 | Disseminated zoster |
| B02.8 | Zoster with other complications |
| B02.9 | Zoster without complications |

**Post-herpetic neuralgia code in HES**

| **ICD code** | **ICD description** |
| --- | --- |
| G53.0 | Postherpectic neuralgia |
| B02.2 | Zoster with other nervous system involvement |

**Appendix C: Definitions used for post-herpetic neuralgia**

The diagnostic algorithms used were an adaptation of the algorithms used in our previous study of risk factors for PHN.^17^ The definition of PHN included diagnosed, probable, or possible PHN, based on a validated algorithm of PHN derived using US administrative data.^18^ Details of these classifications are given in the Table below. All three classifications were used to identify PHN in the linked electronic health data in the present study.

**Classifications for Postherpetic Neuralgia (PHN)**

| **Diagnosed PHN** |
| --- |
| PHN code 90-365 days after zoster, recorded in CPRD or in HES data |
| **Probable PHN** |
| a) Zoster code and a prescription consistent with PHN^1^ on the same day, 90-365 days after zoster |
| b) Non-specific neuralgia or neuropathic pain code, 90-365 days after zoster, with no previous neuralgia/ neuropathic pain code in the 365-14 days before the date of zoster diagnosis  c) New prescription for anticonvulsant, capsaicin or lignocaine patch, 90-180 days after zoster diagnosis |
| d) New prescription for a tricyclic antidepressant (TCA) 90-180 days after zoster diagnosis, with no other indication for the TCA on the day of the prescription, and evidence that the TCA was prescribed for zoster previously |
| e) Zoster code and a pain clinic code on the same day, 90-365 days after zoster |
| **Possible PHN** |
| a) New prescription for strong painkiller, 90-180 days after zoster, with no other indication for the painkiller on the day of the prescription |
| b) New prescription for a TCA, 90-180 days after zoster, with no other indication for the TCA on the day of the prescription |
| c) Pain clinic code 90-365 days after zoster, with no other indication for the pain clinic on the same day |

^1^ Prescription for an anticonvulsant, tricyclic antidepressant, capsaicin cream or lidocaine patch
